# Association between osteoporosis and mortality in Parkinson's disease with mediating effect of hip fractures: a Korean nationwide population-based study

**DOI:** 10.3389/fnagi.2025.1552381

**Published:** 2025-05-21

**Authors:** Yeonju Jin, Bo Kyu Choi, Jong Woo Lee, Jin Yong Hong, Ickpyo Hong, Min Seok Baek

**Affiliations:** ^1^Department of Occupational Therapy, Graduate School, Yonsei University, Wonju, Republic of Korea; ^2^Department of Neurology, Yonsei University College of Medicine, Gangnam Severance Hospital, Seoul, Republic of Korea; ^3^Department of Neurology, Yonsei University Wonju College of Medicine, Wonju Severance Christian Hospital, Wonju, Republic of Korea; ^4^Department of Occupational Therapy, College of Software and Digital Healthcare Convergence, Yonsei University, Wonju, Republic of Korea; ^5^Research Institute of Metabolism and Inflammation, Yonsei University Wonju College of Medicine, Wonju, Republic of Korea

**Keywords:** Parkinson's disease, hip fracture, osteoporosis, mortality, mediating effect

## Abstract

**Introduction:**

This study investigated the association between osteoporosis and mortality in patients with Parkinson's disease (PD) and the mediating role of hip fractures.

**Methods:**

A retrospective cohort study. Data were obtained from the 2009–2019 Korean National Health Insurance Service–National Sample Cohort databases. We extracted both the International Classification of Diseases, 10th Edition code (G20) and PD registration code (V124) to identify patients with PD. A Cox proportional hazards model was used to analyze the association between osteoporosis and mortality. Mediation analyses were performed to estimate the mediating effect of hip fracture between osteoporosis and mortality in patients with PD.

**Results:**

Of the 2,084 patients with PD, 474 (18.5%) were diagnosed with osteoporosis, and 112 (4.4%) experienced hip fractures after PD diagnosis. In unadjusted mediation analysis, the direct effect of osteoporosis on mortality was not significant (β = 0.0309, 95%: confidence interval [CI] −0.0180–0.0798, *p* = 0.2149), whereas the indirect effect of hip fracture was (β = 0.0130, 95% CI 0.0048–0.0212, *p* = 0.0019). Similarly, in the adjusted model controlling for sex, age at diagnosis, and Charlson Comorbidity Index, the direct effect was not significant (β = 0.0011, 95% CI−0.0508–0.0529, *p* = 0.9675), whereas the indirect effect was (β = 0.0061, 95% CI 0.0009–0.0114, *p* = 0.0223).

**Discussion:**

This study elucidated the association between osteoporosis and mortality in patients with PD by highlighting the mediating role of hip fractures. These findings thus underscore the importance of managing osteoporosis in patients with PD.

## 1 Introduction

Parkinson's disease (PD) is a neurodegenerative disease typically characterized by abnormal motor symptoms, including tremors, rigidity, and bradykinesia. PD also affects gait, balance, and postural stability, consequently elevating the susceptibility to fractures (Samii et al., 2004). The prevalence of PD is associated with an increased risk of fractures, with disease severity being linearly associated with fracture risk (Nam et al., 2021; Koo et al., 2023). Fracture risk has been observed across all body parts in patients with PD, with hip fractures presenting the greatest risk (Mühlenfeld et al., 2021). Furthermore, the risk of mortality in PD with hip fractures is twice as high as those without fractures (Schini et al., 2020).

Osteoporosis, a condition characterized by decreased bone mineral density (BMD), bone mass, and alterations in bone structure and strength, presents a potential risk for fractures. PD progression can lead to malnutrition and sarcopenia, which increase the risk of osteoporosis (Torsney et al., 2014). Decreased BMD further exacerbates the susceptibility to hip fractures in patients with PD. The risk of osteoporotic fractures in patients with PD and osteoporosis is nearly double, while the risk of hip fractures is triple those of patients with PD without osteoporosis (Pouwels et al., 2013).

However, a study previously reported that osteoporosis did not significantly affect the risk of hip fractures in patients with PD (Kim et al., 2022). Instead, patients with PD had a higher risk of fractures than those without PD, regardless of their osteoporosis status. Although PD and osteoporosis independently influence facture risk, their relationship at the time of fracture occurrence remains unclear. Furthermore, although clinicians recognize that hip fractures and osteoporosis contribute to mortality among patients with PD, the specific mediating effect of hip fractures on the association between osteoporosis and mortality in patients with PD remains insufficiently explored.

Therefore, this study investigated the association between osteoporosis and mortality in patients with PD and explored the mediating role of hip fractures in the progression from osteoporosis to mortality.

## 2 Materials and methods

### 2.1 Study population

In this retrospective cohort study, we analyzed data obtained from the South Korea National Health Insurance Service–National Sample Cohort (NHIS-NSC) databases. The NHIS operates as a mandatory single-payer healthcare system (http://nhiss.nhis.or.kr). The NHIS-NSC database is a large cohort representing 2% of the entire national population and sampled based on sex, age, income level, and region. The database collects demographic characteristics, diagnoses according to the International Classification of Diseases, 10th Edition (ICD-10), and mortality (Lee et al., 2018; Kim et al., 2020). Furthermore, for rare and intractable diseases, the claims database includes a special code (V-code) designated by the national registration program (Park et al., 2015).

**Figure 1:**
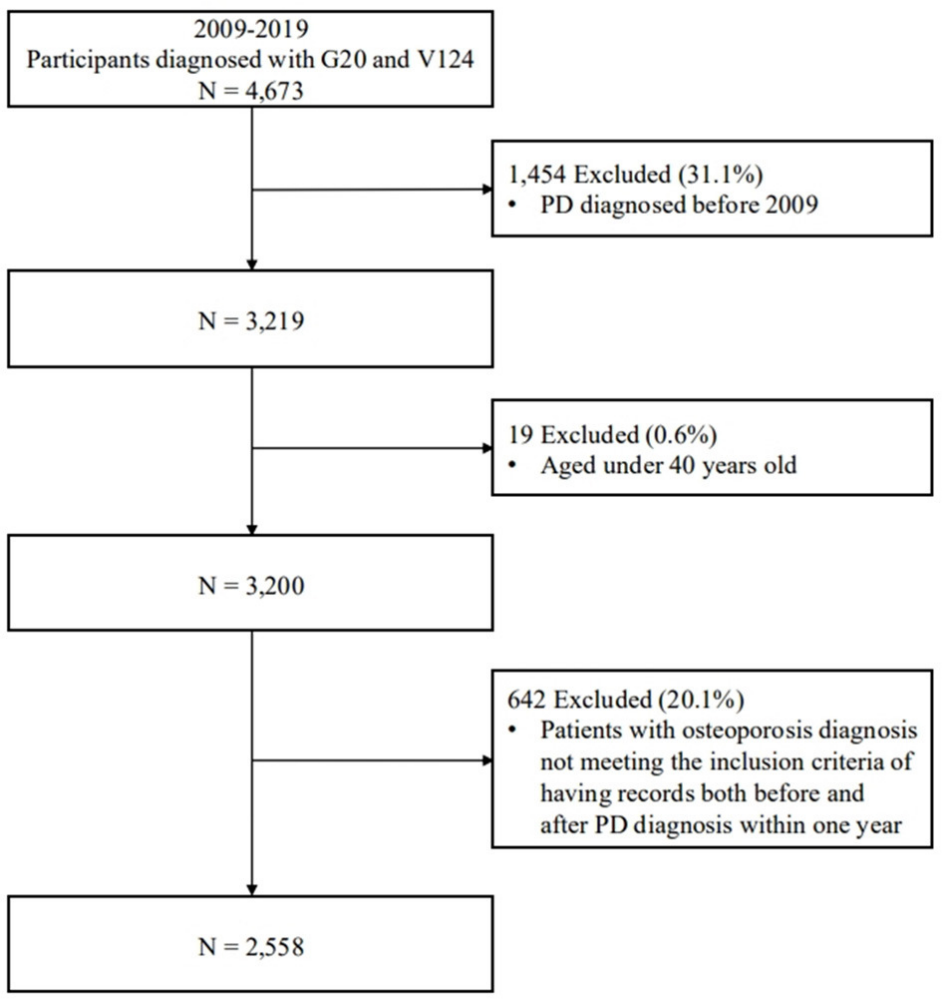
A flow diagram of the participant selection process. The diagram shows inclusion and exclusion criteria, along with the final number of participants categorized according to study requirements. PD, Parkinson's disease.

Using the ICD-10 (G20) and registration V-codes (V124), we identified study subjects registered in the claims database who were diagnosed with PD between January 1, 2009, and December 31, 2019. Patients diagnosed with PD prior to 2009 were excluded from the analysis. Figure 1 shows a flow diagram of the subject selection process.

The NHIS databases are de-identified and publicly available with approval from the National Health Insurance Big Data Department in South Korea, thus waiving the requirement for informed consent. The use of the data was approved by the NHIS Inquiry Commission and the Institutional Review Board (IRB) of Wonju Severance Christian Hospital (IRB number: CR321308). This study was also approved by the IRB of the Yonsei University Mirae Campus number: (IRB number: 1041849-202309-SB-171-01).

### 2.2 Operational definitions

We adopted the operational definitions of osteoporosis, hip fracture, and PD used in previous studies (Koo et al., 2023; Kim et al., 2022; Lee et al., 2018). The diagnosis of osteoporosis was determined by the presence of specific ICD-10 codes (M80, M81, and M82). Considering the nature of osteoporosis, which requires long-term follow up, and the characteristics of claims data, we defined patients with osteoporosis as those who had complete medical records for both pre- and postdiagnosis of osteoporosis within 1 year of PD diagnosis. We defined hip fractures using ICD-10 codes (S72.0, S72.1, S72.2, S72.3, S72.4, S72.7, S72.8, and S72.9). Patients with a history of hip fracture prior to their osteoporosis or PD diagnosis were excluded. The NHIS databases provide information on the year and month of death (National Health Insurance Service, 2024). The date of death was determined based on events that occurred following the diagnosis of PD, and patients who died before the diagnosis of PD were excluded from the study cohort. Mortality status was categorized as “1,” while survival was categorized as “0”.

Demographic characteristics were used as covariates, including age at PD diagnosis, sex, the modified Charlson Comorbidity Index (CCI), and levodopa equivalent daily dose (LEDD). The modified CCI was determined using ICD-10 codes for diagnoses within 1 year before the diagnosis of PD and categorizing the number of comorbidities into three categories (0, 1, 2, or higher) (Quan et al., 2005). The LEDD was calculated at the time of enrollment and subsequently log-transformed to ensure a normal distribution and improve the precision of the mediation analysis.

### 2.3 Statistical analysis

The chi-square test and *t*-test were used to compare the characteristics of patients with PD with and without hip fractures. The Cox proportional hazards model was used to analyze time-to-event data and thus estimate the hazard ratios (HR) for the risk of osteoporosis related mortality in patients with PD. The model was adjusted for relevant covariates, including age, sex, and comorbidities. The estimates were expressed with HR and 95% confidence intervals (CI). Statistical significance was determined using a *p*-value threshold of 0.05.

Mediation analysis was performed to estimate the mediating effect of hip fractures on the association between osteoporosis and mortality by analyzing the covariates (Agler and De Boeck, 2017). In the mediation analysis, the independent variable was osteoporosis (X), the mediation variable was hip fractures (M), and the dependent variable was mortality (Y). A *p* < 0.05 was considered statistically significant. Data management and all statistical analyses were performed using SAS version 9.4 (SAS Institute Inc, 2013).

## 3 Results

### 3.1 Descriptive statistics

In total, 2,558 patients diagnosed with PD between January 1, 2009, and December 31, 2019, were enrolled in this study. Table 1 presents the descriptive statistics comparing patients with (*n* = 474; 18.5%) and without (*n* = 2,084; 81.5%) osteoporosis. Patients with osteoporosis had a higher percentage of hip fractures (*n* = 46, 9.7% vs. *n* = 66, 3.2%; *p* < 0.0001) and a higher proportion of women (*n* = 431, 90.9% vs. *n* = 941, 45.2%, *p* < 0.0001). Patients with osteoporosis showed a higher mean ± SD age at PD diagnosis than those without osteoporosis (74.6 years ± 6.9 vs. 70.2 years ± 9.9, *p* < 0.0001). However, PD severity assessed by LEDD, as well as mortality, did not differ significantly between the two groups.

**Table 1:** Descriptive statistics comparing patients with and without osteoporosis, *n* (%).

**Variables**	**Full sample with PD; *N* = 2,558 (100%)**	**Osteoporosis**		***p***
		**Yes;** ***N*** = **474 (18.5%)**	**No;** ***N*** = **2,084 (81.5%)**	
**Hip fracture**				<0.0001^*^
Yes	112 (4.4)	46 (9.7)	66 (3.2)	
No	2,446 (95.6)	428 (90.3)	2,018 (96.8)	
**Age at PD diagnosis, mean (SD)**	71.0 (9.6)	74.6 (6.9)	70.2 (9.9)	<0.0001^*^
**Log-transformed LEDD, mean (SD)**	3.3 (4.2)	3.5 (4.2)	3.3 (4.2)	0.3032
**Age group**				<0.0001^*^
40–59	319 (12.5)	12 (2.5)	307 (14.7)	
60–69	652 (25.5)	81 (17.1)	571 (27.4)	
70–79	1,107 (43.3)	268 (56.5)	839 (40.3)	
80+	480 (18.8)	113 (23.8)	367 (17.6)	
**Observation period, monthly, mean (SD)**	56.6 (35.5)	53.2 (31.7)	57.4 (36.2)	0.0201^*^
**Sex**				<0.0001^*^
Female	1,372 (53.6)	431 (90.9)	941 (45.2)	
Male	1,186 (46.4)	43 (9.1)	1,143 (54.9)	
**CCI**				<0.0001^*^
0	801 (31.3)	37 (7.8)	764 (36.7)	
1	479 (18.7)	82 (17.3)	397 (19.1)	
≥2	1,278 (50.0)	355 (74.9)	923 (44.3)	
**Mortality**				0.0772
Yes	1,004 (39.3)	203 (42.8)	801 (38.4)	
No	1,554 (60.8)	271 (57.2)	1,283 (61.6)	

CCI, Charlson comorbidity index; LEDD, levodopa equivalent daily dose; PD, Parkinson's disease; SD, standard deviation. ^*^Statistically significant at an alpha level of 0.05.

### 3.2 Cox regression analysis

Table 2 presents the data on the Cox proportional hazards model examining the associations between risk factors and mortality in patients with PD. In Model 1, patients with osteoporosis showed a higher risk of mortality than those without (HR 1.295, 95% CI 1.109–1.512, *p* = 0.0011). In contrast, Model 2, which was adjusted for age, sex, and comorbidities, did not show a significant relationship between osteoporosis and mortality (HR 0.988, 95% CI 0.829–1.177, *p* = 0.8913).

**Table 2:** Cox proportional hazards model for mortality associated with osteoporosis in patients with PD.

**Variables**	**Model 1**		**Model 2**	
	**HR (95% CI)**	***p***	**HR (95% CI)**	***p***
**Osteoporosis**				
No	1		1	
Yes	1.295 (1.109–1.512)	0.0011^*^	0.988 (0.829–1.177)	0.8913
**Sex**				
Female			1	
Male			1.837 (1.601–2.107)	<0.0001^*^
**Age group**				
40–59			1	
60–69			1.559 (1.153–1.108)	0.0039^*^
70–79			2.600 (1.963–3.443)	<0.0001^*^
80+			5.011 (3.737–6.719)	<0.0001^*^
**CCI**				
0			1	
1			2.194 (1.801–2.673)	<0.0001^*^
≥2			2.669 (2.262–3.149)	<0.0001^*^

CCI, Charlson comorbidity index; CI, confidence interval; ^*^Statistically significant at an alpha level of 0.05.

### 3.3 Mediation analysis

Mediation analysis examined the unadjusted mediation effect of hip fractures on the association between osteoporosis and mortality (Supplementary Table 1). The direct (Path c) and total effects of osteoporosis and mortality were not statistically significant. However, osteoporosis was significantly associated with hip fractures (Path a: β = 1.1897, 95% CI 0.7991–1.5804, *p* < 0.0001), and hip fractures were significantly associated with increased mortality (Path b: β = 0.1985, 95% CI 0.1057–0.2913, *p* < 0.0001). Furthermore, the natural indirect effect, representing the mediation effect of hip fractures, was significant (β = 0.0130, 95% CI 0.0048–0.0212, *p* = 0.0019), indicating that hip fractures partially mediate the association between osteoporosis and mortality.

**Figure 2:**
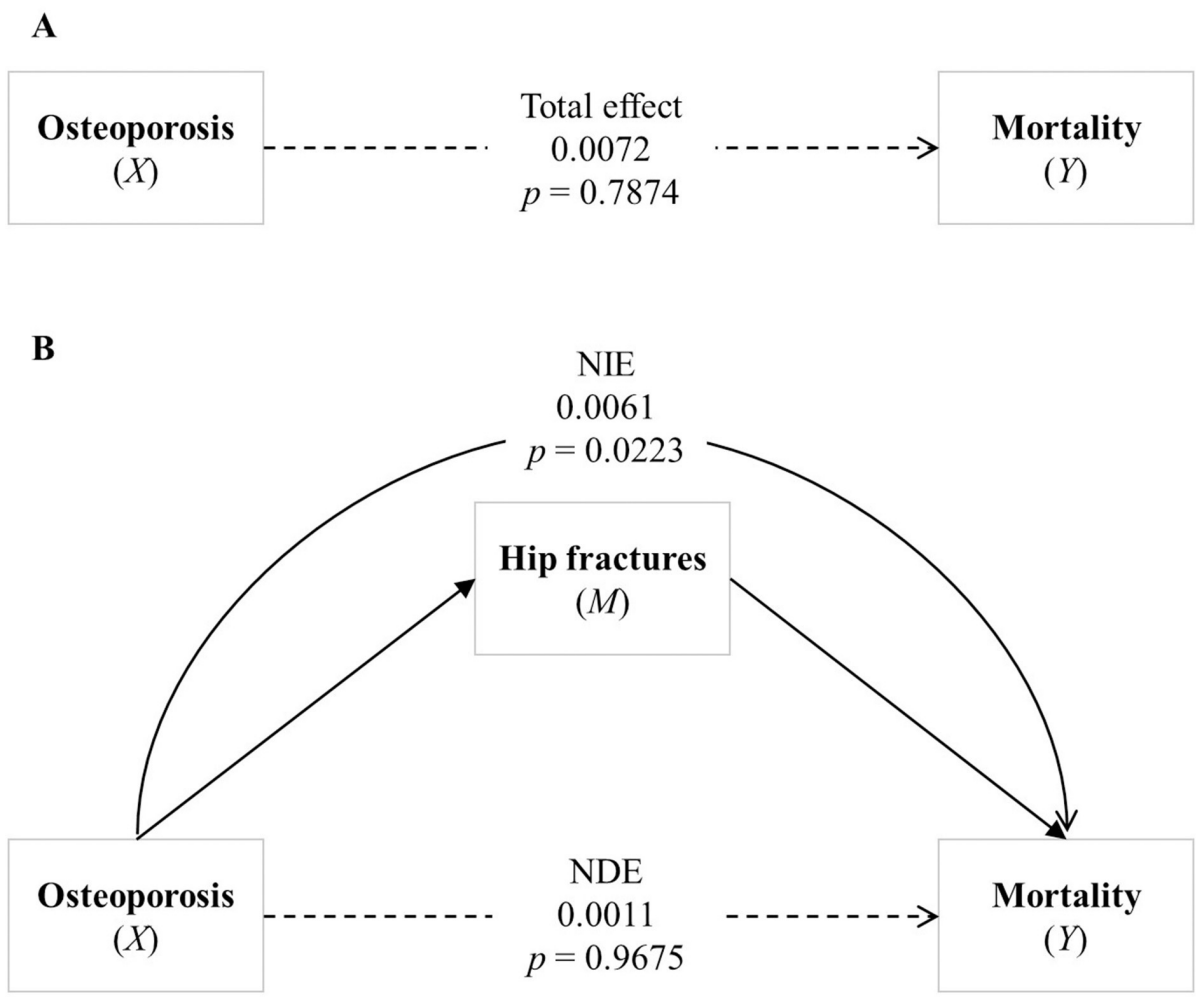
Analysis of the association between osteoporosis and mortality. **(A)** Shows the total effect of osteoporosis on mortality. **(B)** Illustrates the mediating effect of hip fracture between osteoporosis and mortality, highlighting the Natural Direct Effect (NDE) and the Natural Indirect Effect (NIE). Bold lines represent statistically significant paths, while dashed lines represent non-significant paths. NDE, Natural Direct Effect; NIE, Natural Indirect Effect.

Figure 2 illustrates the mediation effect determined by the mediation analysis of hip fractures on the association between osteoporosis and mortality, in the model adjusted for sex, age group, and CCI. Furthermore, the direct and the total effects of osteoporosis and mortality were not significantly associated in the adjusted model (Figures 2A, B, respectively). Osteoporosis remained significantly associated with hip fractures (Path a: β = 0.9527, 95% CI 0.4947–1.4107, *p* < 0.0001), and hip fractures were significantly associated with increased mortality (Path b: β = 0.1516, 95% CI 0.0627–0.2404, *p* = 0.0008; Table 3). The natural indirect effect of hip fractures was significant (β = 0.0061, 95% CI 0.0009–0.0114, *p* = 0.0223), indicating that hip fractures partially mediate the relationship between osteoporosis and mortality, even after adjusting for covariates. These findings remained consistent after further adjustment for PD severity, as assessed by log-transformed LEDD values (Supplementary Table 2).

**Table 3:** Association between osteoporosis and mortality mediated by hip fracture in a covariate-adjusted model.

**Path**	**β**	**95% CI**		***p***
a^*^	0.9527	0.4947	1.4107	<0.0001
b^**†**^	0.1516	0.0627	0.2404	0.0008
c^**‡**^	0.0011	−0.0508	0.0529	0.9675
Total effect	0.0072	−0.0446	0.0590	0.7847
Natural direct effect (NDE)	0.0011	−0.0508	0.0529	0.9675
Natural indirect effect (NIE)	0.0061	0.0009	0.0114	0.0223

Adjusted for sex, age group, Charlson comorbidity index (CCI), confidence interval (CI).

^*^Path a: Association between osteoporosis (yes vs. no) and hip fracture (yes vs. no).

^**†**^Path b: Association between hip fracture (yes vs. no) and mortality (yes vs. no).

^**‡**^Path c: Association between osteoporosis (yes vs. no) and mortality (yes vs. no).

## 4 Discussion

This nationwide cohort study demonstrated the interrelationships among osteoporosis, hip fractures, and mortality in patients with PD. Specifically, our results indicate that hip fractures act as a full mediator in the relationship between osteoporosis and mortality in patients with PD. Thus, osteoporosis in PD is not directly associated with increased mortality but is linked to it only through hip fractures. Our study results can be used as reference in developing strategies aimed at reducing the risk factors for mortality in patients with PD, particularly by addressing the role of hip fractures in the relationship between osteoporosis and mortality.

The unadjusted Cox proportional hazards model revealed that osteoporosis was significantly associated with increased mortality rate in patients with PD (HR = 1.295, *p* = 0.0011). However, the association was no longer significant after adjusting for covariates, such as sex, age, and CCI, (HR = 0.988, *p* = 0.8913). Previous longitudinal studies have shown that male sex, older age at onset, and presence of comorbidities are associated with increased mortality risk (Pinter et al., 2015; Hoogland et al., 2019; Forsaa et al., 2010). Consistent with previous studies conducted in the general population, this study also demonstrated that although the prevalence of osteoporosis was markedly higher in women than in men, the risk of mortality associated with osteoporosis was notably higher in men (Zhang et al., 2024; Lee et al., 2013). They also reported that osteoporosis is not an independent major risk factor for mortality in patients with PD, indicating that interactions with other underlying factors may contribute more significantly to increased mortality (Pinter et al., 2015; Forsaa et al., 2010). Further studies should be conducted to clarify the causal relationship between osteoporosis and mortality in PD and the various associated factors.

Contrary to a previous study using a nationwide database, which reported that osteoporosis does not significantly increase the risk of hip fractures in patients with PD (Kim et al., 2022), our study found that hip fractures fully mediate the relationship between osteoporosis and mortality in PD. Thus, osteoporosis is not directly associated with mortality but is significantly linked to it only through hip fractures. Patients with PD are at high risk for both osteoporosis and hip fractures (Malochet-Guinamand et al., 2015). As PD progresses, various symptoms (e.g., mobility impairment, decreased hand–mouth coordination, dysphagia, and reduced gastrointestinal motility) can lead to malnutrition and sarcopenia. These secondary symptoms are also associated with an increased risk of osteoporosis and reduced BMD, leading to a higher risk of osteoporotic and hip fractures (Torsney et al., 2014; Pouwels et al., 2013). In particular, medication with levodopa in PD is associated with hyperhomocysteinemia, an independent risk factor for osteoporosis, as well as common deficiencies in vitamin B12 and folate (Figueroa and Rosen, 2020). Gao et al. (2015) found a negative correlation between daily levodopa dosage and BMD at the spine and hip in patients with PD. Some studies have shown that bisphosphonates, vitamin D, and calcium therapy can increase BMD and reduce fractures in patients with PD (van den Bos et al., 2012; Cummings et al., 2019).

Furthermore, mediation analysis revealed that hip fractures are significantly associated with increased mortality in PD. A retrospective cohort study among older Medicare beneficiaries in the United States found that patients with PD had a significantly higher adjusted mortality rate (HR = 2.41) after hip/pelvic fractures compared with patients without PD (Harris-Hayes et al., 2014). Nam et al. (2021) reported that patients with PD and hip fractures had twice the mortality rate compared with those without fractures. Another nationwide population-based study in Korea matched patients with and without PD and examined comorbidities associated with mortality in patients with PD (Yoon et al., 2021). In their study, no significant difference in mortality related to hip fractures was observed in patients aged <59 and <80 years. However, a significant association was found in the 60–79 age group. Our study reported results similar to those of previous studies on the increased risk of mortality associated with hip fractures after the onset of PD. Notably, subgroup analysis indicated that the relationship between hip fractures and mortality varied significantly with age (Yoon et al., 2021). These findings emphasize the need for age-specific interventions to reduce hip fracture-related mortality in patients with PD and indicate the necessity for further research to explore other factors influencing this relationship. To this end, interventions such as increasing calcium and vitamin D intake through dietary sources or supplementation in older adults, along with the implementation of fall prevention strategies, may be considered as potential approaches.

This study has several limitations. First, the diagnoses of PD, osteoporosis, hip fracture, and comorbidities were based on ICD codes of the NHIS-NSC database. Therefore, inaccuracies in the claims data may have resulted in disease misclassification. Second, because of the characteristics of claims data, clinical information, including the severity of PD symptoms and cognitive functions, was not included in the data analysis. In addition, clinical parameters such as disease stage, motor subtypes, fall history, nutritional status, and physical function were also unavailable, all of which may influence both fracture risk and post-fracture mortality. Future prospective studies incorporating more granular clinical data are warranted to elucidate the relationship between hip fracture and mortality in patients with PD. The study population should include PD patients with osteoporosis, and regular follow-up assessments should systematically document potential confounding factors, including the severity of parkinsonism. To ensure more precise temporal assessment and stronger causal inference, future studies may need to incorporate regular bone mineral density evaluations, such as dual energy X-ray absorptiometry. Third, we only considered the initial hip fracture that occurred after the onset of PD and osteoporosis for analysis. We did not include subsequent fractures and thus did not analyze details regarding multiple fractures. Additionally, although we focused on hip fracture as the principal diagnosis to assess its mediating effect on mortality, we did not explicitly exclude individuals with co-existing vertebral fractures. Consequently, it is possible that some participants had both hip and vertebral fractures, which may have influenced the observed outcomes. This limits our ability to attribute the mediating effect solely to hip fractures. Future research should aim to analyze different types of osteoporotic fractures—such as hip and vertebral fractures—both independently and in combination, to better understand their respective and interactive contributions to mortality risk in patients with Parkinson's disease. Given that ICD-10 codes M80, M81, and M82 represent different subtypes of osteoporosis, which may reflect varying disease severity or underlying causes, future studies should consider analyzing these subgroups separately.

This study emphasizes that osteoporosis is associated with increased risk of hip fractures, highlighting the indirect role of hip fractures in the mortality of patients with PD and concurrent osteoporosis. For these patients, thus, interventions should include not only the prescription of medications to treat and prevent osteoporosis but also to the implementation of measures minimizing the risk of hip fractures. Our findings underscore the importance of managing the risk factors of osteoporosis related to disease progression and medication use in PD, emphasizing the need for proactive strategies for hip fracture prevention.

## Data Availability

The data analyzed in this study is subject to the following licenses/restrictions: the datasets presented in this article are not readily available because they are derived from medical claims provided by the South Korea National Health Insurance Service, and restrictions apply to their availability. Requests to access these datasets should be directed to National Health Insurance Service, https://nhiss.nhis.or.kr.
