# Supplementary material for: Association between osteoporosis and mortality in Parkinson's disease with mediating effect of hip fractures: a Korean nationwide population-based study
Source: Front Aging Neurosci. 2025 May 21;17:1552381. doi: 10.3389/fnagi.2025.1552381 (PMC12133770; doi:10.3389/fnagi.2025.1552381)
Supplement: Supplementary file 1 [file Table_1.docx]

Supplementary Material

# Supplementary Tables

**Supplementary Table 1. Association between osteoporosis and mortality mediated by hip fracture in an unadjusted model**

| Path | $\beta$ | 95% CI | | *p* |
| --- | --- | --- | --- | --- |
| a**^*^** | 1.1897 | 0.7991 | 1.5804 | <.0001 |
| b**^†^** | 0.1985 | 0.1057 | 0.2913 | <.0001 |
| c**^‡^** | 0.0309 | -0.0180 | 0.0798 | 0.2149 |
| Total Effect | 0.0439 | -0.0049 | 0.0927 | 0.0779 |
| Natural Direct Effect (NDE) | 0.0309 | -0.0180 | 0.0798 | 0.2149 |
| Natural Indirect Effect (NIE) | 0.0130 | 0.0048 | 0.0212 | 0.0019 |

*Note.* CI, confidence interval.

**^*^**Path a: Association between osteoporosis (yes vs. no) and hip fracture (yes vs. no)

**^†^**Path b: Association between hip fracture (yes vs. no) and mortality (yes vs. no)

**^‡^** Path c: Association between osteoporosis (yes vs. no) and mortality (yes vs. no)

**Supplementary Table 2. Association between osteoporosis and mortality mediated by hip fracture in a covariate-adjusted model including log-transformed LEDD**

| Path | $\beta$ | 95% CI | | *p* |
| --- | --- | --- | --- | --- |
| a**^*^** | 0.8364 | 0.3956 | 1.2772 | 0.0002 |
| b**^†^** | 0.1179 | 0.0331 | 0.2027 | 0.0064 |
| c**^‡^** | 0.0045 | -0.0470 | 0.0560 | 0.8644 |
| Total Effect | 0.0090 | -0.0424 | 0.0604 | 0.7322 |
| Natural Direct Effect (NDE) | 0.0045 | -0.0470 | 0.0560 | 0.8644 |
| Natural Indirect Effect (NIE) | 0.0045 | 0.0001 | 0.0089 | 0.0452 |

*Note.* Adjusted for sex, age group, Charlson comorbidity index (CCI), and levodopa equivalent daily dose (LEDD). CI, confidence interval.

**^*^**Path a: Association between osteoporosis (yes vs. no) and hip fracture (yes vs. no)

**^†^**Path b: Association between hip fracture (yes vs. no) and mortality (yes vs. no)

**^‡^** Path c: Association between osteoporosis (yes vs. no) and mortality (yes vs. no)
